# A palette of site-specific organelle fluorescent thermometers

**DOI:** 10.1016/j.mtbio.2022.100405

**Published:** 2022-08-19

**Authors:** Xiao Liu, Takeru Yamazaki, Haw-Young Kwon, Satoshi Arai, Young-Tae Chang

**Affiliations:** a Center for Self-assembly and Complexity, Institute for Basic Science (IBS), Pohang, Gyeongbuk, 37673, South Korea; b Department of Chemistry, Pohang University of Science and Technology (POSTECH), Pohang, Gyeongbuk, 37673, South Korea; c WPI Nano Life Science Institute, Kanazawa University, Kakuma-machi, Kanazawa, 920-1192, Japan

**Keywords:** Fluorescence, Temperature, Molecular rotor, Organelle, Thermometers

## Abstract

Intracellular micro-temperature is closely related to cellular processes. Such local temperature inside cells can be measured by fluorescent thermometers, which are a series of fluorescent materials that convert the temperature information to detectable fluorescence signals. To investigate the intracellular temperature fluctuation in various organelles, it is essential to develop site-specific organelle thermometers. In this study, we develop a new series of fluorescent thermometers, Thermo Greens (TGs), to visualize the temperature change in almost all typical organelles. Through fluorescence lifetime-based cell imaging, it was proven that TGs allow the organelle-specific monitoring of temperature gradients created by external heating. The fluorescence lifetime-based thermometry shows that each organelle experiences a distinct temperature increment which depends on the distance away from the heat source. TGs are further demonstrated in the quantitative imaging of heat production at different organelles such as mitochondria and endoplasmic reticulum in brown adipocytes. To date, TGs are the first palette batch of small molecular fluorescent thermometers that can cover almost all typical organelles. These findings can inspire the development of new fluorescent thermometers and enhance the understanding of thermal biology in the future.

## Graphical abstract

Thermo Greens (TGs) are the first batch of fluorescent thermometers that can cover almost all typical organelles. The FLIM thermometry shows that each organelle experiences a distinct temperature increment depending on the distance away from the heat source. TGs are further demonstrated in the quantitative imaging of the heat production at different organelles in brown adipocytes.

**Figure:**
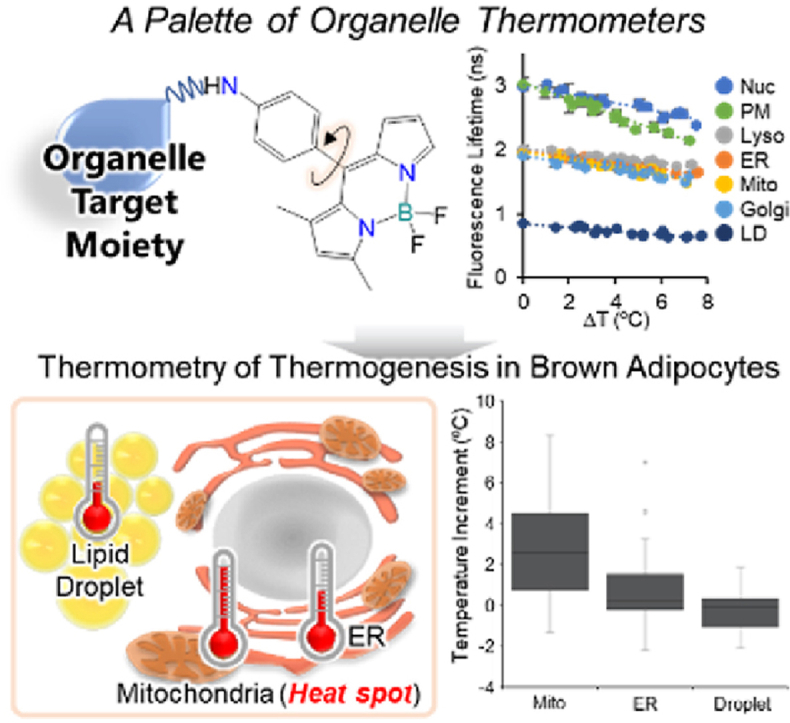

## 1 Introduction

Temperature is a fundamental and significant physical parameter for cellular systems. The environmental temperature can significantly influence the diffusion, metabolism, differentiation and proliferation of cells [[1], [2], [3], [4]]. Furthermore, the change in the temperature of cells is not only caused by the environment but also generated from the metabolic heat related to biological processes [[5], [6], [7]]. In recent years, considerable interest has been focused on temperature fluctuation and heterogeneity at the single-cell level [[8], [9], [10]]. Therefore, mapping the micro-temperature in various organelles at a subcellular scale is of great importance for understanding various biological events.

Fluorescent thermometers are one of the powerful means that allows the read-out of a minor temperature change as the alteration of fluorescence signals such as fluorescence intensity, anisotropy and lifetime [11]. To investigate the intracellular temperature variation, different types of thermometers have already been developed, including polymers, quantum dots, nanodiamonds, small-molecule fluorescent probes and fluorescent proteins [9,10,[12], [13], [14], [15], [16], [17], [18], [19], [20]]. Among them, molecular fluorescent probes possess the advantage of even distribution to any type of cells derived from various species. Previously, to explore a molecular fluorescent thermometer, we conducted an unbiased screening of a fluorescent dye library (DOFL: Diversity Oriented Fluorescence Library) [13,21]. Consequently, we could find out a boron-dipyrromethene (BODIPY) derivative with a temperature sensitivity of 3.9%/°C (intensity) and 26 ​ps/°C (lifetime) in live cells [13,22]. It should also be noted that the dye possesses a targeting ability to the endoplasmic reticulum (ER). Finally, we demonstrated the dye in organelle thermometry of heat production at ER and reported the first molecular fluorescent temperature sensor, ER thermo yellow (termed ETY in this paper) [13]. Beyond that, Kriszt and his colleagues generated ERthermAC through the improvement of ETY in terms of photostability and demonstrated the qualitative imaging of heat production in brown adipocytes [9]. During the same screening of DOFL, another rosamine dye based-fluorescent thermometer, Mito Thermo Yellow (MTY), was also found for mitochondrial thermometry with a temperature sensitivity of 2.7%/°C [20]. Using MTY, Chrétien et al. demonstrated that the local temperature increase at mitochondria was closely related to respiratory processes [8].

Since previous biochemical studies have pointed out that mitochondria and ER acted as heat spots in cells, these molecular thermometers supported these hypotheses with the visual evidence of intracellular temperature variations, particularly in thermogenic cells such as brown adipocytes and skeletal muscle [9,23,24]. However, organelle thermometry is limited to ER and mitochondria due to the poor usability of targeted fluorescent thermometers. There are still many other organelles remaining unexplored, such as the Golgi body, lipid droplets, lysosome, nucleus and plasma membrane.

This study reports the synthesis and application of a new series of fluorescent thermometers, Thermo Greens (TGs), covering almost all typical organelles. To our knowledge, TGs are the first series of small molecular thermometers for organelle-targeted thermometry at various organelles, including the ones that have never been focused on viewpoints of thermal biology. Using fluorescence lifetime imaging microscopy (FLIM), TGs validated the feasibility of organelle-specific thermometry. Finally, the palette application of TGs is expanded for the thermal imaging of heat production in different organelles in brown adipocytes using FLIM. We believe that this palette of new fluorescent thermometers is critical for exploring unknown questions in biology.

## 2 Results and discussion

### 2.1 Probe design and characterization

**Scheme 1:**
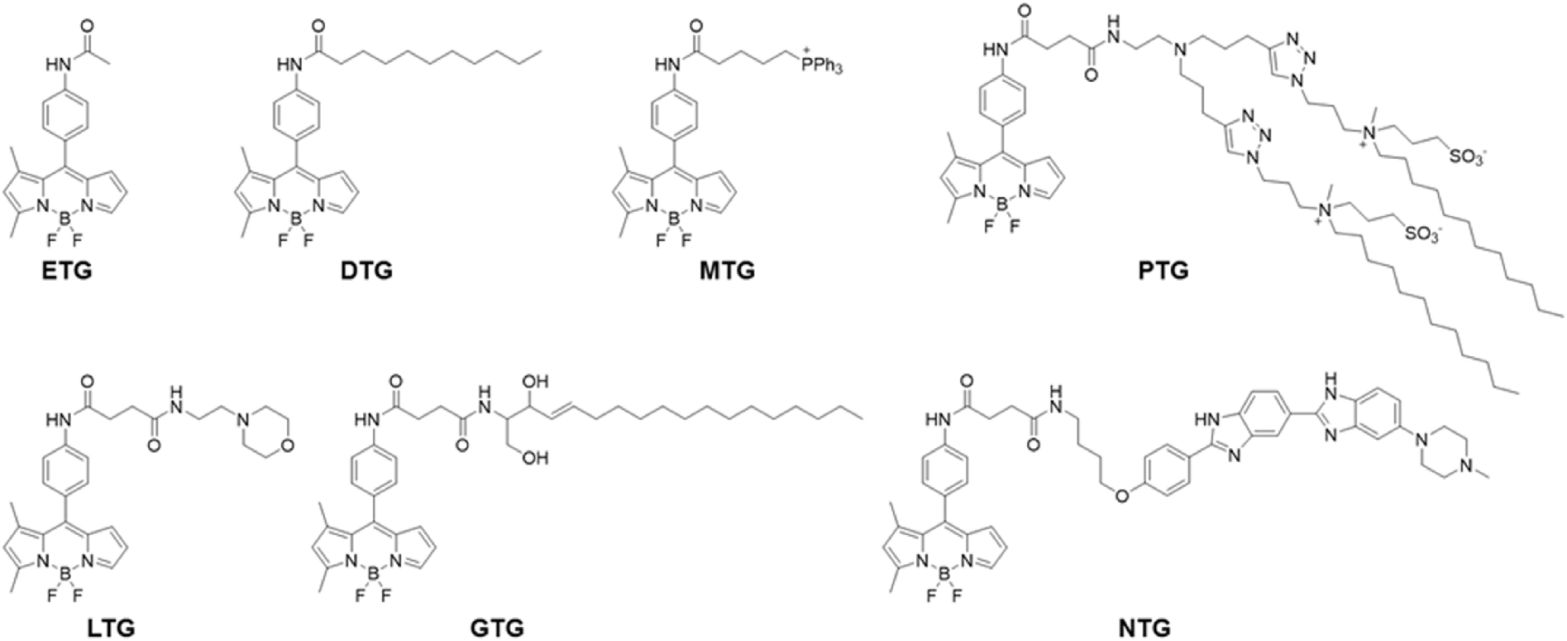
Molecular structures of Thermo Greens. ETG: ER Thermo Green; DTG: Lipid Droplet Thermo Green; MTG: Mitochondria Thermo Green; LTG: Lysosome Thermo Green; PTG: Plasma Membrane Thermo Green; GTG: Golgi Thermo Green; NTG: Nucleus Thermo Green.

As mentioned above, albeit ETY and MTY were developed using different backbones of dyes respectively, more attention has been paid to a BODIPY-based ETY because of its higher sensitivity than the other rosamine-based one. A key determinant for the probe design of fluorescent thermometers is the physicochemical property of BODIPY as a molecular rotor [25]. According to our previous study, the viscosity sensitivity of BODIPY rotors was reversely correlated to the transformation energy barrier from the planar structure to the butterfly-like form [25]. By modifying BODIPY cores, the unsymmetrical BODIPY (named ETG in this work) possessed the lowest energy barrier, and has been reported to be the most viscosity-sensitive BODIPY so far [26]. In other words, ETG can be a promising scaffold to sense the change in the physical microenvironment as the detectable fluorescence signal with high sensitivity. Therefore, it is expected that ETG (ER Thermo Green) may also exhibit good sensitivity to the intracellular temperature change [[25], [26], [27]]. Starting from the structure of ETG, an expanded series of green fluorescent thermometers covering almost all typical organelles were synthesized (Scheme 1). Various organelle-targeting motifs were added to the unsymmetrical BODIPY structure, such as n-undecanoyl group (Lipid Droplet Thermo Green, DTG) [28], triphenylphosphonium group (Mitochondria Thermo Green, MTG) [29], 4-(2-aminoethyl)morpholine group (Lysosome Thermo Green, LTG) [30], 3-(dodecyl(methyl)amino)propane-1-sulfonate (Plasma Membrane Thermo Green, PTG) [31], d-sphingosine group (Golgi Thermo Green, GTG) [29] and Hoechst 33,258 (Nucleus Thermo Green, NTG) [32]. The new batch of site-specific organelle thermometers was named Thermo Greens (TGs).

The addition of organelle-targeting motifs did not significantly change the fundamental photophysical properties of the organelle thermometers. The absorption and emission wavelengths of the newly developed thermometers are all around 496 ​nm and 512 ​nm, which is very similar to the properties of the mother compound, ETG (Table 1 and Fig. S1). This excitation and emission window pretty much matches the most common filter set of fluorescent microscopes (FITC or GFP), which has never been done by previous yellow color-based MTY and ETY. Other than that, the absorption spectrum of NTG displayed a second peak at 353 ​nm due to the existence of the Hoechst motif. The quantum yields of TGs only slightly varied from 1.28% to 1.74%. Overall, TGs displayed very similar photophysical properties with relatively low quantum yields.

**Table 1:** Photophysical properties of Thermo Greens.

Compound	λabs[nm]^[a]^	λem[nm]^[a]^	Φ^[^a), b)^,^^b^^]^
ETG	496	511	1.28%
DTG	496	513	1.37%
MTG	496	512	1.51%
LTG	496	512	1.42%
GTG	496	512	1.57%
PTG	496	512	1.74%
NTG	353/495	511	1.38%

a) Fundamental photophysical properties of Thermo Greens were measured in DMSO.

b) Quantum yields were calculated utilizing fluorescein (Φ ​= ​0.91 in 0.1 ​N NaOH) as a standard reference.

### 2.2 Fluorescence lifetime-based temperature sensitivity evaluation of TGs in live cells

**Fig. 1:**
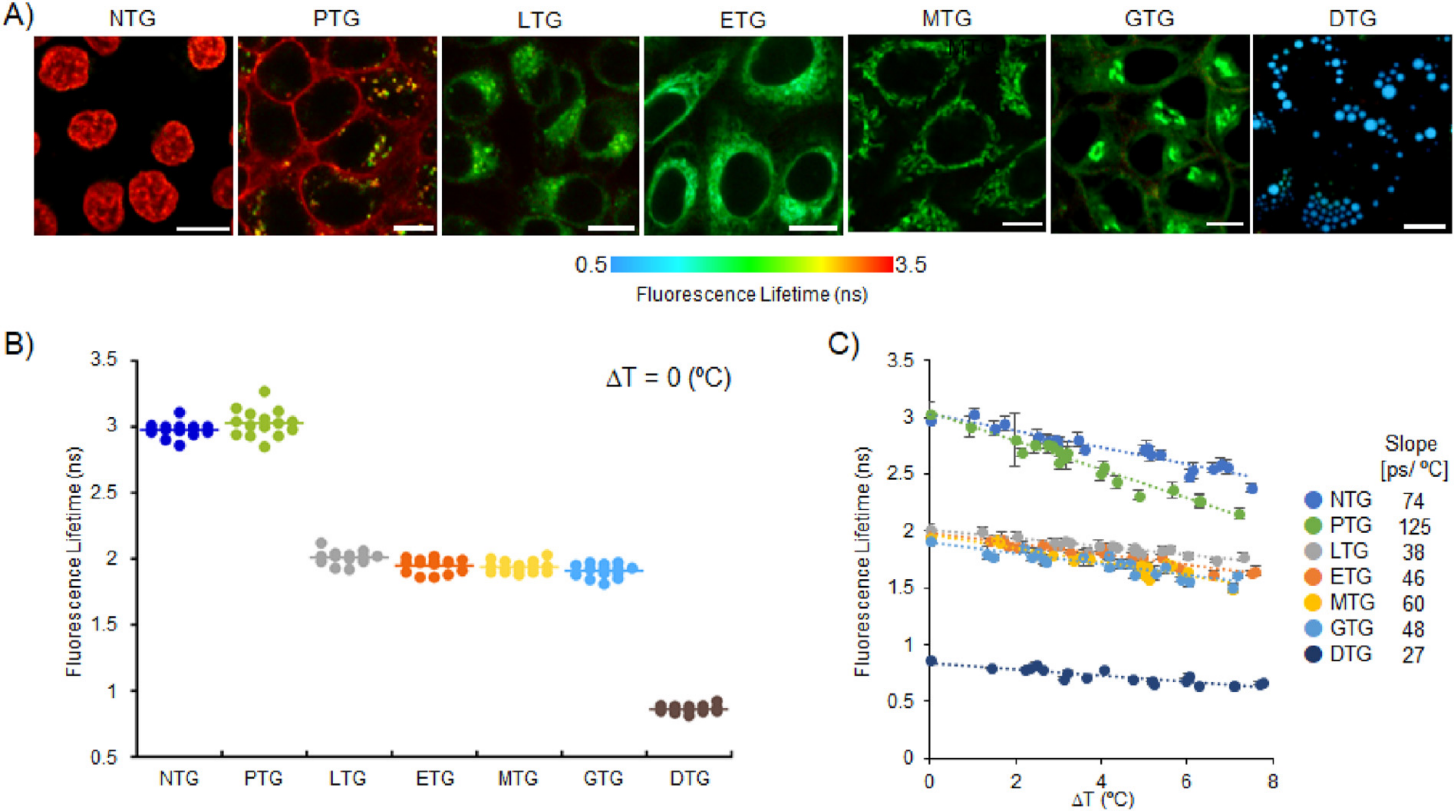
Calibration curves of TGs in live cells. (A) FLIM images of TGs in HeLa cells (only DTG in brown adipocytes). Scale bars: 10 ​μm. (B) The evaluation of fluorescence lifetimes of TGs in live cells at base temperature (37 ​°C, ΔT ​= ​0). The line indicates the average of 10 ​cells. The statistical analysis was performed by Tukey's multiple comparisons. P ​< ​0.001: PTG vs. DTG, GTG, MTG, ETG, and LTG. NTG vs. DTG, GTG, MTG, ETG, and LTG. DTG vs. LTG, ETG, MTG, and GTG. LTG vs. GTG. p ​< ​0.05: LTG vs. MTG. NS: ETG vs. LTG, GTG, and MTG. PTG vs. NTG. MTG vs. GTG. (C) The calibration curves of fluorescence lifetime against temperature increments in HeLa cells and brown adipocytes (DTG). Each data point represents means ​± ​SD (n ​= ​19–20) obtained from the exponential curve fitting.

Firstly, the organelle-specificity of newly developed TGs was examined, showing that all thermometers are located in the targeted organelles as expected (Fig. S2). In addition, all thermometers displayed minimal cytotoxicity to cells (Fig. S3). Next, we investigated the fluorescence lifetimes of TGs at each organelle under the basal condition (ΔT ​= ​0) (Fig. 1A). As shown in Fig. 1B, the fluorescence lifetime of PTG was much longer than DTG. It could be reasoned that the rigidity of the plasma membrane is much higher than that of the droplets with randomly oriented lipids, implying that PTG prefers to exist in the planar form as a molecular rotor under the rigid environment, leading to a longer fluorescence lifetime than that of DTG [25,33]. On the contrary, DTG is more likely to exist in the butterfly form under the fluidic conditions inside the droplets, resulting in a shorter lifetime [25]. Also, a recent study on the liquid order in the membranes mentioned that intracellular membranes such as ER, Golgi, mitochondria and lysosome are more flexible than that of plasma membranes [34]. Assuming that these TGs are located in organelle membranes due to the hydrophobicity of the dyes, it should also be compatible with results that the lifetimes of LTG, ETG, MTG and GTG were shorter than PTG under the basal conditions. Unlike the others, NTG does not retain in the membrane but sticks to the condensed DNA due to the Hoechst motif, which would prevent the free motion of the rotor, promoting the planar structure with a longer lifetime.

Because the microenvironment surrounding each TG differs in the organelle, it could be imagined that the temperature sensitivity of TGs in the test tube is different from that in an organelle (Fig. S4). This is because the temperature response is governed by the property of the medium in each organelle [13,20]. Therefore, we evaluated the temperature dependency of the fluorescence lifetime of TG at each organelle location under the live-cell condition and define the slope as *in situ* temperature sensitivity. To quantify the relationship between temperature and lifetime of TGs, the fluorescence lifetime of each TG compound was plotted versus temperature variations in cells (Fig. 1C, the method to prepare the calibration curve was described in Fig. S5-**7**). As it turns out, the fluorescence lifetime of all TG compounds is inversely correlated with temperature increments. In similar with the other types of previous fluorescent thermometers, the slopes were also fitted well with linear curves within a narrow range of physiological temperature [11]. Here, it should also be noted that a BODIPY rotor molecule is known to be sensitive to both viscosity and temperature [26,35]. When the fluorescence signal is altered in response to the temperature increment, it is quite challenging to separate the temperature effect from the viscosity effect because the temperature increment is also accompanied by the viscosity change at the same time. Thus, the obtained slopes to represent the temperature sensitivity contain the totality of temperature and temperature-induced viscosity change effects (the detail of the sensing mechanism will be addressed in the discussion part).

Among seven compounds, PTG exhibited the highest intracellular temperature sensitivity, which is 125 ​ps/°C, followed by NTG (74 ​ps/°C) and MTG (60 ​ps/°C, Fig. 1C). ETG and GTG possessed similar temperature sensitivities, ranging from 46 ​ps/°C to 48 ​ps/°C, while LTG displayed slightly lower sensitivity, 38 ​ps/°C. DTG exhibited the lowest temperature sensitivity in live cells, which is 27 ​ps/°C. As with the discussion regarding the basal conditions (Fig. 1B), it is assumed that these obvious differences in the slopes also reflect the feature of molecular environments surrounding TGs. For instance, previous studies revealed that the liquid order of plasma membranes is close to the artificial lipid membranes (sphingomyelin/cholesterol) as a model of the liquid ordered (L_o_) phase [36]. On the other hand, intracellular membranes such as ER, Golgi, lysosome and mitochondria constitute less L_o_ phase and thereby endow more fluid states like liquid disordered (L_d_) phases [34,37]. Apart from that, it is likely that L_o_ phase has a greater range of the change in membrane fluidity in response to the temperature change than L_d_ phase [38]. Because the ease of the rotation of the substituent in TGs was governed by the temperature-induced fluidity change, the slope of PTG would be steeper than the others such as LTG, ETG, MTG and GTG. Although the theory of L_o_-L_d_ phases could not be applied to the fluidity of lipid droplet, ordered structures to restrict the movement of DTG rarely exists inside the oil, resulting in the most gradual slope. Regarding NTG, the temperature increment promotes the dissociation of NTG from DNA and increases the population of the freely-moving NTG. Accordingly, we speculated that this thermodynamic element contributes to the relatively higher steep slope than the other TGs.

**Fig. 2:**
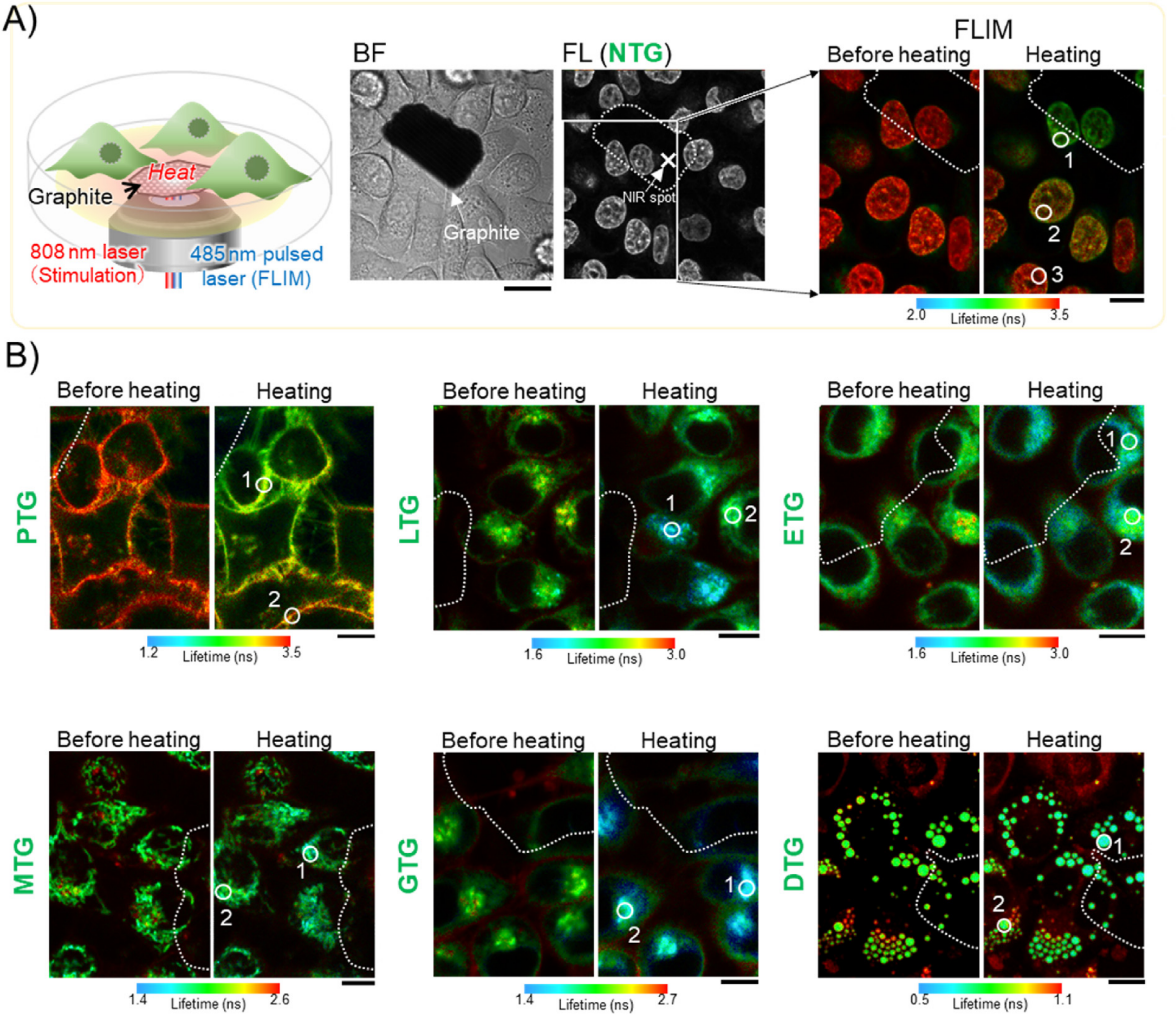
Visualization of temperature gradient created by an 808 ​nm laser. (A) Schematic illustration of a microscopic system where an 808 ​nm laser is coupled with a graphite flake to produce the local heat (left panel). The bright field and fluorescence images of NTG in HeLa cells were shown in the center panel. Scale bar: 20 ​μm. FLIM images of NTG were shown before and after heating (for each 30 ​s) at 11 ​mW. The temperature increments at different positions (ROI1-3) were analyzed from the calibration curve (fluorescence lifetime vs. ΔT). 1) 8.2 ​± ​0.5 ​°C, 2) 3.6 ​± ​0.4 ​°C, 3) 2.2 ​± ​0.5 ​°C. Scale bar: 10 ​μm. (B) Similar to NTG as shown in A), temperature gradients generated by an 808 ​nm laser illumination were imaged in HeLa cells using the other TGs (only DTG in BAT). The temperature increments at different positions (ROI 1–2) were analyzed from the calibration curve. PTG: 1) 4.9 ​± ​0.4 ​°C, 2) 1.7 ​± ​0.5 ​°C, LTG: 1) 8.1 ​± ​0.6 ​°C, 2) 6.3 ​± ​0.4 ​°C, ETG: 1) 6.3 ​± ​0.6 ​°C, 2) 4.3 ​± ​0.8 ​°C, MTG: 1) 6.8 ​± ​0.4 ​°C, 2) 2.6 ​± ​0.4 ​°C, GTG: 1) 7.5 ​± ​0.4 ​°C, 2) 5.8 ​± ​0.4 ​°C, DTG: 1) 5.8 ​± ​0.3 ​°C, 2) 2.1 ​± ​0.4 ​°C. Scale bars: 10 ​μm.

Furthermore, we examined the ability of TGs to detect the temperature gradient that occurs at a microscopic level. As shown in Fig. 2A, during microscopic observation with FLIM, an 808 ​nm laser was illuminated to the graphite flake located at the center of the observation area through an objective lens according to the previous literature [13]. The laser irradiation during 15 ​s increases the temperature of the local heat spot, and then the generated heat is transmitted from graphite to the surrounding cells, causing a temperature gradient from the heating center (high temperature) to the surrounding area (low temperature). For reference, photothermal heating during such a short time scarcely caused critical damage to HeLa cells unless the temperature increment does not reach approximately 11 ​°C from the base temperature of 37 ​°C, which was supported by previous literature [39]. Using such a microscopic set-up, ETG, MTG, LTG, GTG, PTG and NTG were evaluated in HeLa cells, while DTG was tested in brown adipocytes due to its large lipid droplets. Upon a NIR laser irradiation, all TGs displayed significant decreases in fluorescence lifetime (Fig. 2A and B). In addition, those ROIs with higher temperature increases showed even lower lifetime values than those far away from the heating center. Therefore, TGs can be successfully utilized to sense the intracellular temperature gradient at the micro-scale. Compared to intensity-based fluorescent thermometers, FLIM-based TGs are more accurate because they are independent of the probe concentration, the amplitude of excitation laser power, and focus drifts. All newly developed fluorescent thermometers work pretty well inside live cells and can be used to map the intracellular micro-temperature gradient quantitatively. To our knowledge, it is the first time to achieve temperature visualization in lipid droplets, lysosomes, the Golgi apparatus, plasma membrane and the nucleus with small molecular probes.

### 2.3 Thermal imaging of heat production in brown adipocytes

**Fig. 3:**
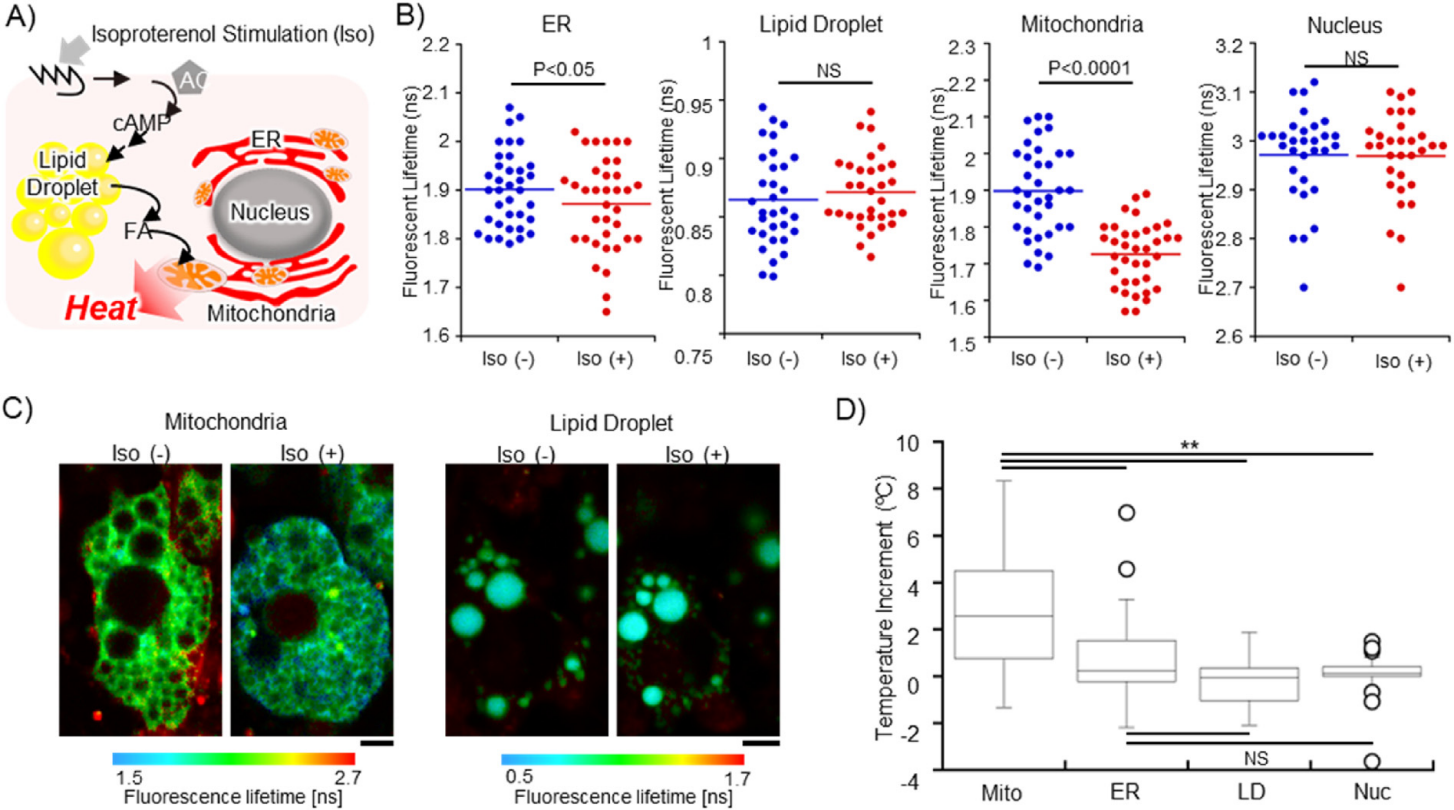
Thermal imaging of heat production in brown adipocytes using FLIM. (A) Schematic illustration of heat production in brown adipocytes. (B) The FLIM analysis of the change in fluorescence lifetime upon the addition of isoproterenol (Iso). The data set was analyzed by paired *t*-test at ER (p ​= ​0.0357), Lipid Droplet (NS), Mitochondria (p ​< ​0.0001), and Nucleus (NS). (C) Visualization of heat production at mitochondria and lipid droplet of brown adipocyte. Scale bar: 5 ​μm. (D) The box plot of the temperature increment at each organelle, analyzed by Tukey's multiple comparisons. The temperature increment was obtained from the same data set with (B) and the calibration curve. Mito, ER, LD and Nuc represent Mitochondria, Endoplasmic Reticulum, Lipid droplet and Nucleus, respectively. The line indicates the median. ∗∗p ​< ​0.001. The number of ROIs is 31–36 (the number of cells is 19–29).

Finally, the applicability of TGs was evaluated regarding whether they can detect physiological heat production or not. The heat production in a thermogenic cell, such as brown adipocytes, has already been investigated at a single cellular level by other methods except for fluorescent thermometry [40,41]. Even though the local temperature increment at the cellular level in different types of cells is still controversial [42,43], thermometry of thermogenesis in a single brown adipocyte was repeated successfully due to the massive amount of heat from the biological process. Starting with the adrenergic stimulation with an agonist like isoproterenol (Iso), cyclic adenosine monophosphate (cAMP) is generated, followed by the activation of protein kinase A (PKA), which in turn releases free fatty acids (FFAs) from lipid droplets and activates the uncoupler protein (UCP1) at the inner membrane of mitochondria [44]. Consequently, it depolarizes the mitochondrial membrane and accelerates mitochondrial respiration, resulting in heat generation (Fig. 3A) [[45], [46], [47]]. Though it is evident that the main heat source exists in mitochondria, it is still unknown how the heat is propagated at the subcellular level. Regarding positions for thermometry, ER and lipid droplets were chosen as well as mitochondria because they are closely located in the vicinity of mitochondria at the subcellular scale. Also, the nucleus was examined as a location that is relatively far from the heat source. Therefore, MTG, ETG, DTG and NTG were utilized to investigate the temperature increment in different organelles. Prior to thermometry in brown adipocytes, it was proven that their fluorescence lifetime could respond to the temperature change reversibly (Fig. S8). Afterward, the thermometry with FLIM was performed before and after the adrenergic stimulation with Iso. In mitochondria, the average fluorescence lifetime of MTG displayed a dramatic decrease from 1.90 ns to 1.73 ns upon the addition of Iso (Fig. 3B and C), indicating that the temperature increment could be detected clearly (P ​< ​0.0001). In addition, the fluorescence lifetime of ETG decreased from 1.90 ns to 1.87 ns, which is also supported by the effective statistical analysis (P ​< ​0.05) (Fig. 3B). Previously, the heat production generated from mitochondria in brown adipocytes could be detected at ER qualitatively by an intensity-based ERthermAC, which is compatible with FLIM-based thermometry with ETG [9]. Therefore, the heat produced in mitochondria may transfer to the adherent ER membrane, leading to the temperature increment at ER [9]. Furthermore, DTG and NTG were also exploited to monitor the temperature change in lipid droplets and nucleus during thermogenesis. Interestingly, both DTG and NTG did not exhibit noticeable lifetime change (Fig. 3B–D and Fig. S9). In the case of NTG, it was reasoned that the heat could not be transmitted to relatively distant locations from the mitochondria. On the contrary, we hypothesized that the lipid droplet could also experience heat initially because the lipid droplets were covered with dense mitochondria and possessed a smaller thermal conductivity (0.135 Wm^−1^K^−1^) than water (0.61 Wm^−1^K^−1^) [48]. We assumed that the reason for the opposite result is that the heat generated from the mitochondria was not sufficient to warm up the micron-sized lipid droplet but enough to heat the nano to submicron-sized ER. In the end, using the calibration curve (Fig. S7), the actual temperature could be estimated, indicating that the temperature increment at mitochondria (2.8 ​± ​2.7 ​°C) was significantly higher than at ER (0.64 ​± ​1.8 ​°C) and the others (LD; −0.24 ​± ​1.0 ​°C, Nuc; −0.03 ​± ​0.87 ​°C) (as mean ​± ​SD, Fig. 3D). These studies are the first case to track heat propagation in a single brown adipocyte. Comparing with the average of temperature measurement accuracy of MTG (±0.55 ​°C), ETG (±0.65 ​°C), DTG (±0.48 ​°C) and NTG (±0.73 ​°C), we ascertained that the temperature variations observed at each organelle were not due to the artifact from the limitation of the measurement (Fig. S7). One possibility is that the temperature variation of mitochondrial heat production should be implicated in the heterogeneity of mitochondrial functions [49]. In fact, it has been pointed out that individual mitochondrion has a unique function even within the same cell [50]. On top of that, in the case of the thermometry at ER, the heat that each ER experiences varies from the different distances from the heat spot of mitochondria, which could also be a reason for the uneven temperature variation.

At the end of this section, the sensing mechanism of TGs is discussed more. More specifically, in order to clarify which TGs can respond to temperature or viscosity predominantly, the lifetimes of TGs were investigated in different ratios of ethylene glycol-glycerol (EG-Gly) mixtures at two different temperatures (Fig. S4). As it turns out, TGs displayed a good response against viscosity as a common viscosity fluorescence probe. Interestingly, even at a different temperature, the viscosity response of TGs was almost the same trend while the temperature effect was negligible. TGs were likely to sense “temperature-induced viscosity change” mainly in the test tube at least. From a different angle, the energy barrier of the probe for the rotation existing at the S1 excited state (Ea) is thought to be a key element to differentiate viscosity and temperature effect [26]. Namely, as the Ea value is higher, the proportion of the temperature effect increases more besides the viscosity effects. That implied that the Ea values of TGs rarely affect the slope in the calibration in the test tube. However, because the Ea value might also be altered by the surrounding medium in live cells, the argument to separate these effects in live cells still remains challenging. Therefore, we could not avoid that the temperature sensitivity of a fluorescent thermometer is defined as the totality of temperature and temperature-induced viscosity change in the fluorescence signal.

The above discussion regarding the sensing mechanism of TGs gives a precaution to the potential users for these probes. We always need to assume that “only” viscosity changes occur in the biological events without the temperature increments. To our knowledge, in the case of mitochondrial thermogenesis, the drastic alteration of lipid compositions has not been reported so far and thereby the viscosity issue was negligible. Thus, the results in the thermometry could address the heat production quantitatively in this paper.

## 3 Conclusion

In general, we have developed a new series of fluorescent thermometers, Thermo Greens, that can cover almost all typical organelles. TGs successfully visualized intracellular temperature changes in multiple organelles at the single-cell level through FLIM measurements. For a long time, mitochondria and ER have been recognized as the only heat source organelles in thermogenic cells. The expanded usability of organelle-specific thermometers would contribute to discovering unknown heat sources at the cellular level. Furthermore, it has been a hot topic regarding how a cell can sense the temperature changes and transduce the signaling events [51]. The precise thermometry at different locations enables unveiling the novel temperature sensing mechanism in the future. We do believe that our work will inspire the understanding of thermal biology and significantly accelerate the discovery of new organelle-targeting fluorescent thermometers for widespread applications.

## 4 Experimental section/methods

### 4.1 Chemicals and methods

All the chemicals and solvents were purchased from Sigma Aldrich, Alfa Aesar, MERCK, Acros, TCI, Combi-blocks or Samchun and used without further purification. Normal phase purifications were carried out using Merck Silica Gel 60 (particle size: 0.040–0.063 ​mm, 230–400 mesh). HPLC purification was performed on Prep. HPLC (Shimazu) with a PDA detector. Purification method, unless indicated, gradient solvent system was water: acetonitrile (ACN) (90:10 to 0:100) with 0.1% formic acid in a run time of 60 ​min; C18(2) Luna column (5 ​μm 100A, 250 ​× ​21.2 ​mm) were used for purification. 1H NMR and 13C NMR spectra were recorded on a Bruker Avance 500 ​MHz and 850 ​MHz NMR spectrometer. Chemical shifts were expressed in parts per million (ppm). All photophysical studies were performed in SpectraMax®M2e spectrophotometer (Molecular Devices) instrument and the obtained data were analyzed using Microsoft Office Excel and Origin 8.5.

### 4.2 Quantum yield measurements

All fluorescent probes were firstly prepared in DMSO at 1 ​mM concentration and then diluted with solvents before tests. All samples contained 1% DMSO unless otherwise directed. Quantum yields for all probes were calculated by Equation (1), utilizing fluorescein in 0.1 ​N NaOH excited at 475 ​nm (Φ ​= ​0.91) as the standard [52,53]. $\Phi_{f}^{i}$ and $\Phi_{f}^{s}$ respectively represented the quantum yields of the sample and standard. F represented the integrated emission of the fluorescence spectrum, n represented the refractive index of the solvents, and f represented absorption factor (f ​= ​1-10^−A^, where A represented absorbance) at the excitation wavelength selected for sample and standard. The emission area was integrated from 495 ​nm to 650 ​nm.

(1) $$\Phi_{f}^{i}\;=\;\left(\frac{F^{i}}{F^{s}}\right)\;\left(\frac{f_{s}}{f_{i}}\right)\;\left(\frac{n_{\text{i}}^{2}}{n_{\text{s}}^{2}}\right)\;\Phi_{f}^{s}$$

### 4.3 Cell viability tests

Various concentrations of probes were added to HeLa cells in a 6-well plate. The plate was then incubated for 6 ​h and 12 ​h in the incubator. After incubation, cells were collected and stained with SYTOX Red (Thermo Fisher) for 15 ​min. And then cells were immediately read on flow cytometry (CytoFLEX SRT, Beckman Coulter, USA) using 638-nm excitation and a 660-nm filter.

### 4.4 Cell culture

HeLa cell was cultured in Dulbecco's modified eagle's medium (DMEM) (ThermoFisher) supplemented with fetal bovine serum (10%) and penicillin-streptomycin. For imaging studies, cells were cultured in a 3.5 ​cm glass-based dish at 37 ​°C, 5% CO_2_. The murine brown adipocytes derived from differentiation of the established WT-1 brown preadipocytes were also used (hereafter brown adipocyte). A procedure for the culture, including the differentiation step, was followed by the previous literature [54].

### 4.5 Staining procedures for the colocalization test

The colocalization test of each organelle thermometer was performed using a corresponding tracker such as ER-Tracker™ Red, BODIPY TR ceramide, SYTOTM61 red fluorescent nucleic acid, MitoTracker Deep Red FM, Cell Mask Orange, and LysoTracker Red DND-99 (ThermoFisher Scientific). Except for the lipid droplet thermometer (DTG), each organelle thermometer, a tracker, and Hoechst 33,342 were added to pre-warmed DMEM in the glass bottom dish (HeLa cells) at 800, 500 and 500 ​nM as a final concentration. Then, it was incubated at 37 ​°C, 5% CO_2_ for 30 ​min. In the case of DTG, the same procedure was applied for murine brown adipocytes using Nile red as a tracker. For the colocalization study, fluorescence images were taken with a confocal microscope (FV1200, Olympus) in three different channels at sequential mode such as Hoechst 33,342 (emission filter set: 430–455 ​nm; laser: 405 ​nm), FITC (emission filter set: 490–540 ​nm; laser: 473 ​nm) for an organelle thermometer, and Texas red (emission filter set: 575–675 ​nm; laser: 561 ​nm) for a tracker.

### 4.6 Fluorescence lifetime imaging (FLIM) of temperature gradient in live cells

The DMSO stock solution of each TG (1 ​mM) was added to the pre-warmed DMEM in the glass bottom dish (HeLa cell or WT-1 brown adipocyte) at 500 ​nM as a final concentration and incubated at 37 ​°C, 5% CO_2_ for 30 ​min. Then, the graphite flake powder (98 ​μm, M199.95, NSC) as a photothermal material was placed on the dish. The FLIM (rapidFLIMHiRes with MltiHarp 150 Time-Correlated Single Photon Counting (TCSPC) unit, PicoQuant) images were taken every 1.66 ​s (512 x 512 pixels) using a 485 ​nm pulsed laser. The FITC emission filter set (520/35 Bright Line HC) was used for imaging of TGs. All data of fluorescence lifetime were analyzed by SymPhoTime 64 (PicoQuant). The fluorescence decay of each TG was fitted with double exponential functions. Through all experiments, the intensity-weighted average fluorescence lifetime was obtained as the following Equation (2);

(2) $$\tau_{int}=\left({\text{A}}_{1}{\tau_{1}}^{2}+{\text{A}}_{2}{\tau_{2}}^{2}\right)/\left({\text{A}}_{1}\tau_{1}+{\text{A}}_{2}\tau_{2}\right)$$,

where ${\text{A}}_{1}$ and $\tau_{1}$ represent the amplitude and the fluorescence lifetime of each component. During microscopic observation, an 808 ​nm laser (100 ​mW, the laser core size: 50 ​μm, FiberLabs Inc.) was illuminated to the graphite flake located at the center of the observation area (10 ​μm) through an oil immersion objective lens (PLAPON 60×, NA ​= ​1.42). During the timelapse experiments, an 808 ​nm laser was illuminated for 60 ​s. The laser power was adjusted from 4.4 to 11 ​mW while the harsh temperature increment was avoided. The open-close timing of the shutter of an 808 ​nm laser was controlled by the IR-LEGO system (IR-LEGO-100/mini/E, 808 ​nm). The temperature increment (ΔT) at each ROI during heating was analyzed according to the calibration curve. How to prepare the calibration curve (fluorescence lifetime vs. ΔT) was described in the detail in Supporting Information.

### 4.7 FLIM analysis of heat production in brown adipocyte

Similar to the procedure mentioned above, the brown adipocyte was stained with an organelle thermometer (MTG, NTG, DTG, and ETG) at 500 ​nM as the final concentration. The FLIM images before and 50 ​min after adding isoproterenol (final conc. 1 ​mM) were taken and the fluorescence lifetime was analyzed according to the double exponential fitting. All microscopic experiments were carried out at 37 ​°C.

## Credit author statement

**Xiao Liu**: Conceptualization, Methodology, Software, Validation, Formal analysis, Investigation, Writing – original draft, Visualization. **Takeru Yamazaki**: Conceptualization, Methodology, Software, Validation, Formal analysis, Investigation, Writing – original draft, Visualization. **Haw-Young Kwon**: Investigation, Methodology, Software, Investigation. **Satoshi Arai**: Conceptualization, Methodology, Resources, Writing – review & editing, Visualization, Supervision, Project administration, Funding acquisition. **Young-Tae Chang**: Conceptualization, Methodology, Resources, Writing – review & editing, Visualization, Supervision, Project administration, Funding acquisition.

## Declaration of competing interest

The authors declare that they have no known competing financial interests or personal relationships that could have appeared to influence the work reported in this paper.
