# Supplementary material for: A palette of site-specific organelle fluorescent thermometers
Source: Mater Today Bio. 2022 Aug 19;16:100405. doi: 10.1016/j.mtbio.2022.100405 (PMC9434161; doi:10.1016/j.mtbio.2022.100405)
Supplement: Multimedia component 1 [file mmc1.docx]

Supporting Information

A Palette of Site-Specific Organelle Fluorescent Thermometers

Xiao Liu ^a,b,†^, Takeru Yamazaki ^c,†^, Haw-Young Kwon ^a^, Satoshi Arai ^c,*^, Young-Tae Chang ^a,b,*^

^a^Center for Self-assembly and Complexity, Institute for Basic Science (IBS), Pohang, Gyeongbuk 37673, Korea

^b^Department of Chemistry, Pohang University of Science and Technology (POSTECH), Pohang, Gyeongbuk 37673, Korea

^c^ WPI Nano Life Science Institute, Kanazawa University, Kakuma-machi, Kanazawa 920-1192, Japan

^†^These authors contributed equally.

*Corresponding authors. Email: [ytchang@postech.ac.kr](mailto:ytchang@postech.ac.kr); [satoshi.arai@staff.kanazawa-u.ac.jp](mailto:satoshi.arai@staff.kanazawa-u.ac.jp)

**Table of Contents**

1. Figure S1. Normalized absorption and emission spectrum of Thermo Greens.

2. Figure S2. The colocalization tests of organelle-specific TGs and commercial tracers.

3. Figure S3. Effect of TGs on cell viability.

4. Figure S4. Temperature and viscosity responses of TGs in vitro.

5. Figure S5. The calibration curve of ETY (ER thermo yellow).

6. Figure S6. The flowchart of how to prepare the calibration curves of TGs in live cells.

7. Figure S7. Calibration curve of TGs (fluorescence lifetime vs. ΔT).

8. Figure S8. Reversibility tests of organelle thermometers.

9. Figure S9. Thermal imaging of heat production in brown adipocytes using FLIM.


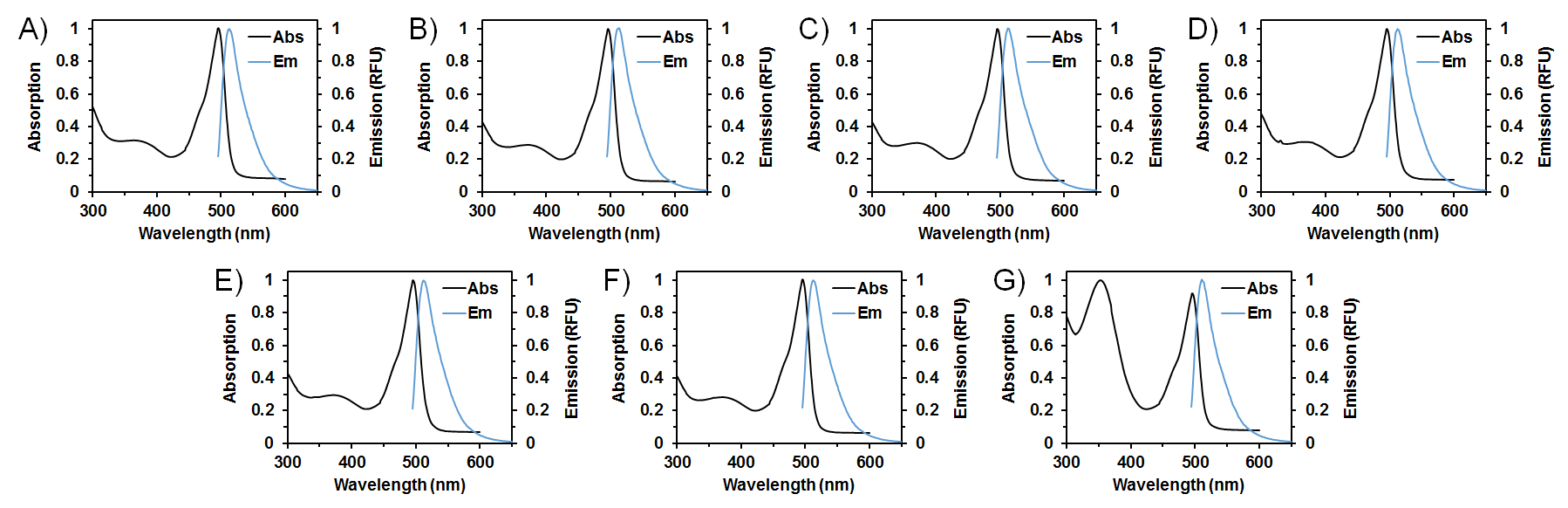


**Figure S1.** Normalized absorption and emission spectrum of Thermo Greens. (A) ETG, (B) DTG, (C) MTG, (D) LTG, (E) GTG, (F) PTG and (G) NTG. Measured in DMSO, 10 μM, λex = 475 nm.


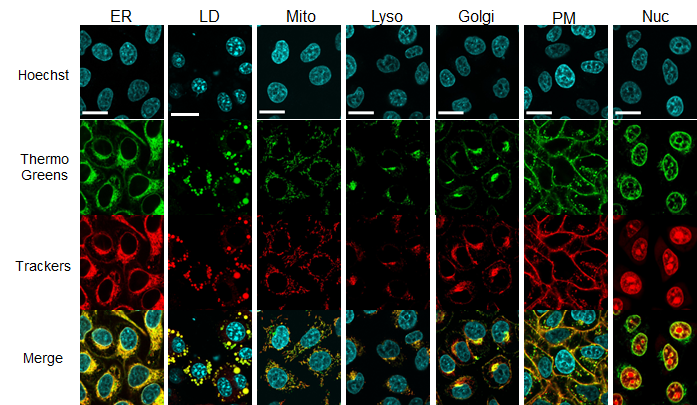


Figure S2. The colocalization tests of organelle-specific TGs and commercial tracers. Scale bar, 20 μm.


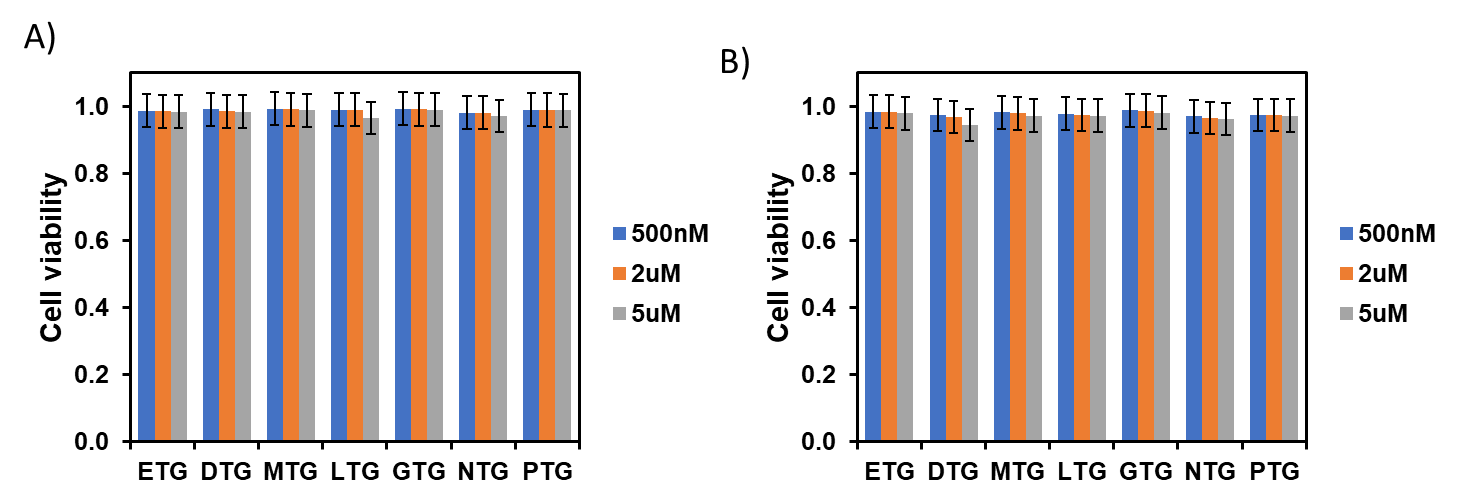


Figure S3. Effect of TGs on cell viability. The viability of HeLa cells after treatment of different concentrations of organelle-specific TGs for (A) 6h and (B) 12h.


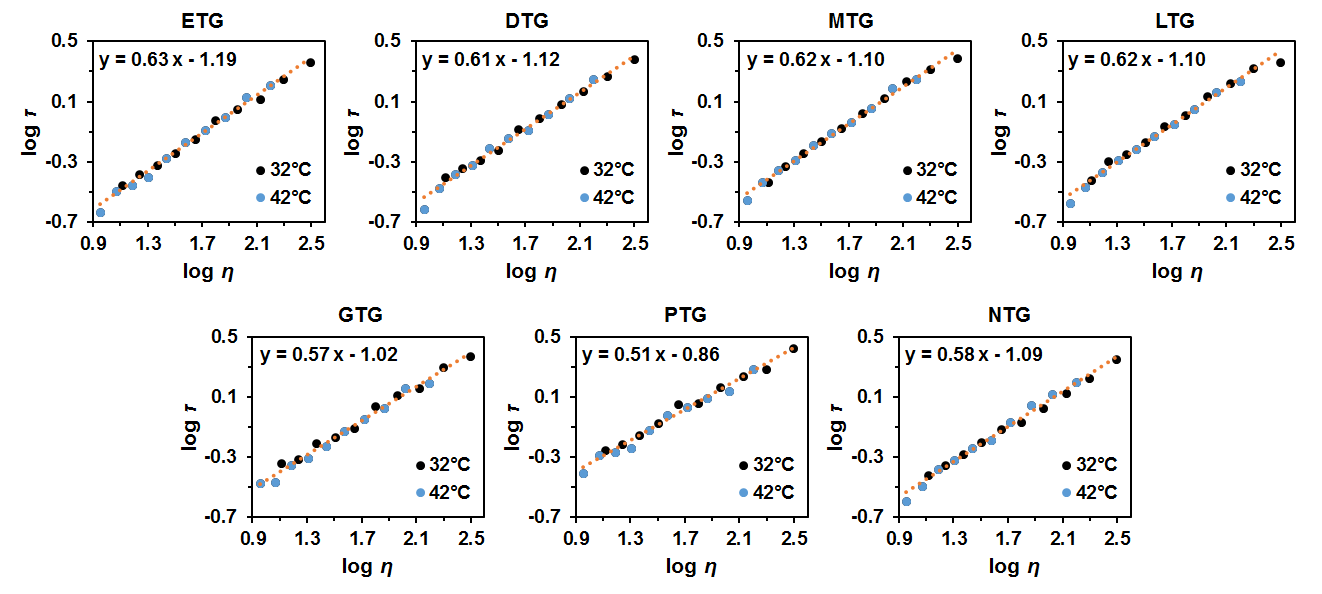


Figure S4. Temperature and viscosity responses of TGs in vitro. The slope was calculated by the equation (1), log τ = C + x log η, reflecting the relationship between fluorescence lifetime (τ) and solvent viscosity (η) at different temperatures. Measured in ethylene glycol-glycerol mixtures, 10 μM probe.


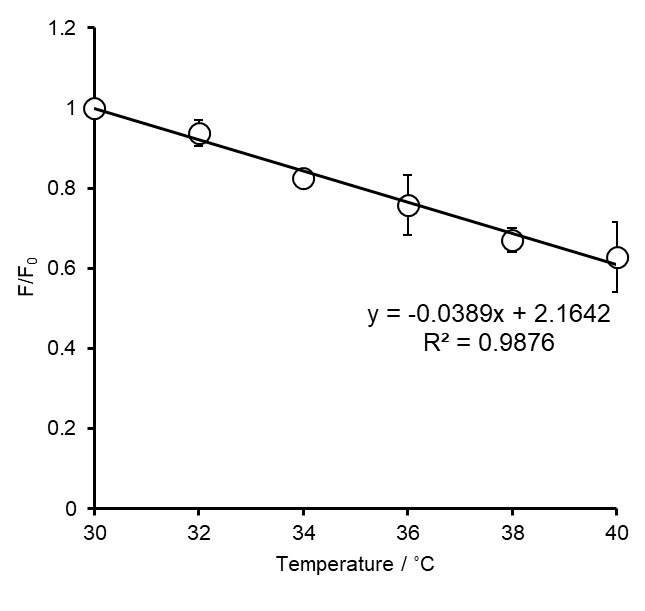
Figure S5. The calibration curve of ETY (ER thermo yellow). The normalized intensity (F/F_0_), where the fluorescence intensity was normalized to that at 30 ˚C, was plotted against different temperatures. The temperature in the dish was controlled by the chamber equipped with the confocal microscope. Each data points represent means ± SD (number of cells were 5).


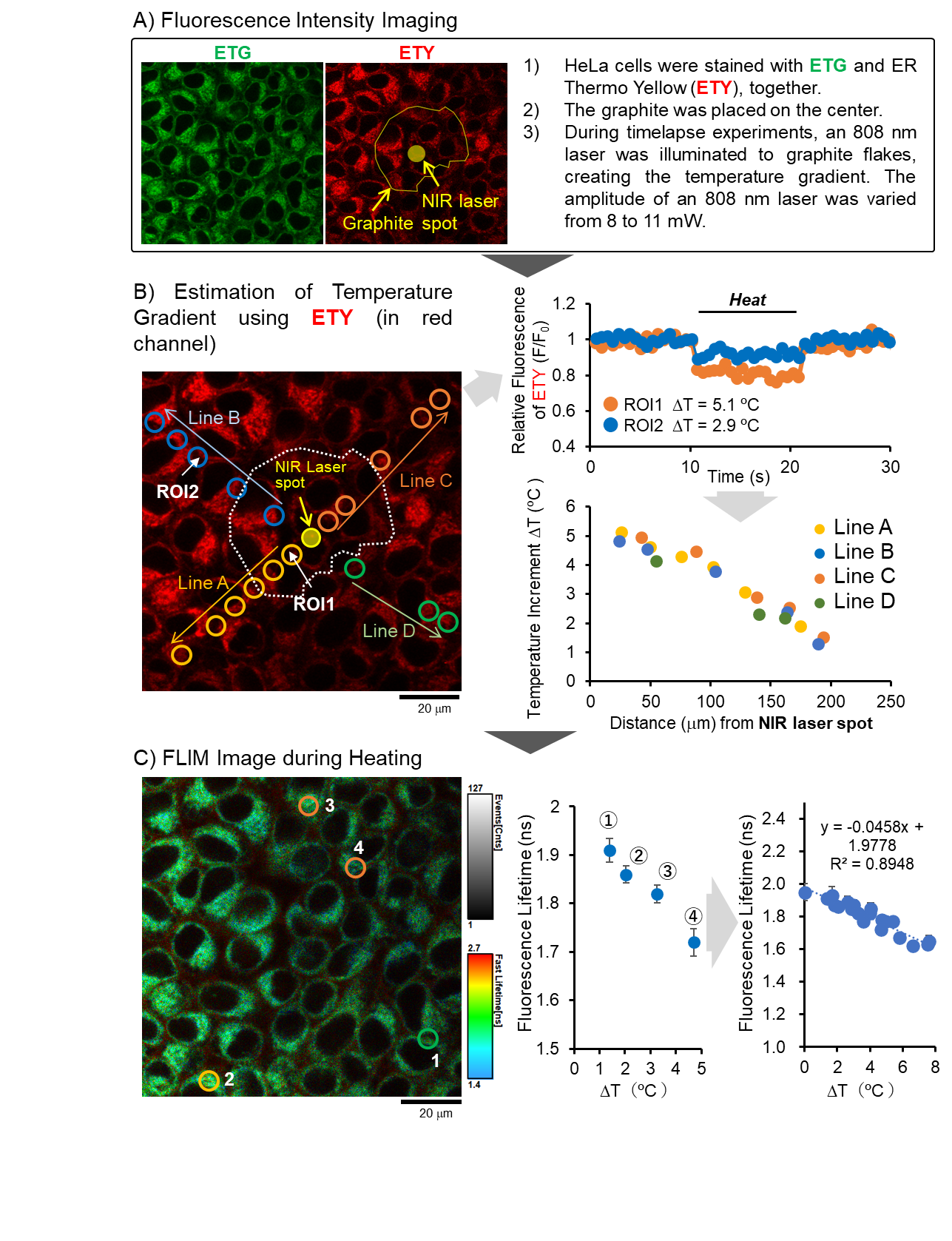


Figure S6. The flowchart of how to prepare the calibration curves of TGs in live cells. (A) HeLa cells were stained with each TG (500 nM) and ER Thermo Yellow (ETY) (500 nM) together. To create the temperature gradient at a microscopic scale, the graphite flake was placed on the dish. While the timelapse imaging of TG and ETY simultaneously was performed using a confocal fluorescence microscope (FV1200, Olympus), an 808 nm laser was illuminated to the flake for 10 seconds to produce the local heat. The amplitude of an 808 nm laser was adjusted from 8 to 11 mW so that it cannot cause the harsh temperature increment to induce cell death. (B) During the illumination of an 808 nm laser, the fluorescence of ETY declined in response to the temperature increment, showing the step-like pattern (the top right panel). From the depth, the temperature increment could be analyzed because the intensity-based temperature sensitivity of ETY was already reported previously as 3.9 %/°C.[6] (for this study, the calibration curve was taken again as seen in Figure S5). As shown in the analysis of ROI1 and 2 (white circles), the degree of the temperature increment depends on the distance between the heat spot and ROIs. More precise analysis showed the temperature gradient which was generated by an 808 nm laser illumination (Line A-D, the bottom light panel). (C) After the intensity-based analysis of the temperature gradient, under the same conditions with the above such as an 808 nm laser power, the duration of heating and the heating location, FLIM imaging of TG was performed using a 485 nm pulse laser (rapidFLIMHiRes with MltiHarp 150 Time-Correlated Single Photon Counting (TCSPC) unit, PicoQuant). The accumulated FLIM images during 10 sec heating were analyzed, providing the fluorescence lifetime values. The calculation method of the fluorescence lifetime was described in Experimental Sections in the main manuscript. For example, the fluorescence lifetimes at different ROIs in the FLIM image were analyzed firstly (ROI1-4 in the left panel). Next, the temperature increments at ROI1-4 were estimated using the result of the temperature gradient analysis of ETY (at the same locations corresponding to the FLIM image) as mentioned above. Then, the fluorescence lifetime was plotted versus temperature increments (the middle panel). The same experiments were repeated at least three times using independent dishes. Finally, the calibration curve was obtained in the right panel. According to this method, the calibration curves of the other TGs were obtained in HeLa cells (only DTG in brown adipocytes), as seen in Figure S7.


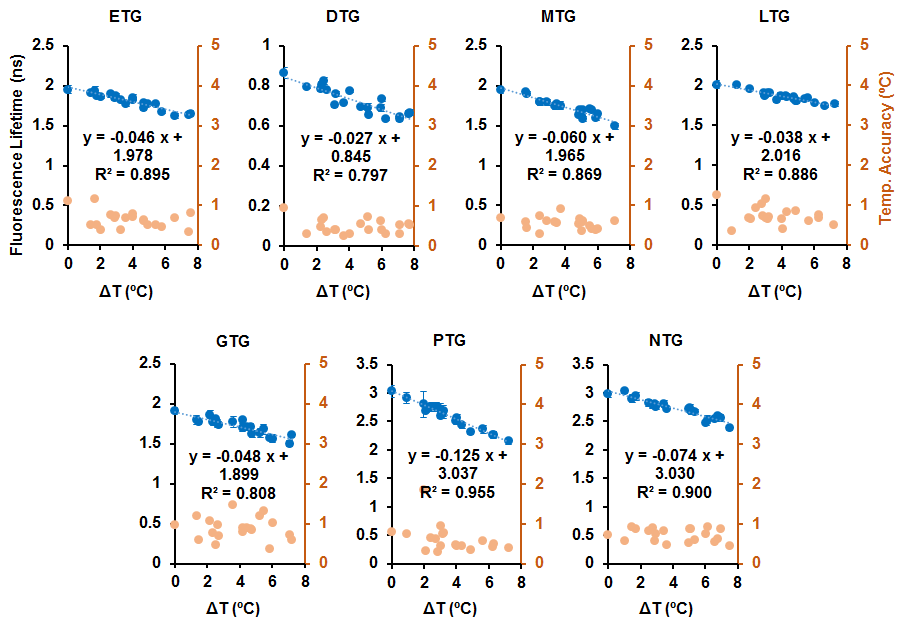


Figure S7. Calibration curve of TGs (fluorescence lifetime vs. ΔT). The base temperature was 37 °C. Each data point represents means ± SD. The second axis shows the accuracy of the temperature measurement with FLIM, which is obtained from the SD value divided by the temperature sensitivity (ps/ °C). The number of plots (=the number of ROI) was 19 in PTG and MTG, and 20 in NTG, GTG, LTG, DTG and ETG. The averages of the accuracy of temperature measurement (means ± SD) are calculated to be 0.65 ± 0.21 °C (ETG), 0.55 ± 0.14 °C (MTG), 0.73 ± 0.16 °C (NTG), 0.48 ± 0.18 °C (DTG), 0.88 ± 0.27 °C (GTG), 0.62 ± 0.34 °C (PTG), 0.45 ± 0.14 °C (LTG).


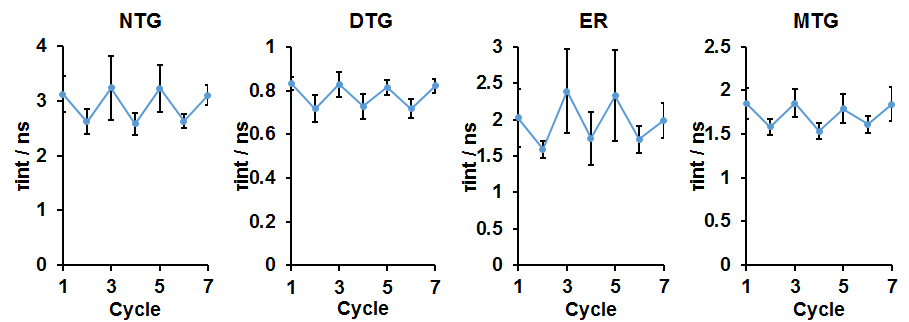


Figure S8. Reversibility tests of organelle thermometers. The ones that were used for monitoring the heat production of brown adipocytes were investigated in terms of the reversibility of fluorescence lifetime. The heat cycle was repeated three times and then the fluorescence lifetime was evaluated. Each data represents mean ± SD (cell numbers = 10).


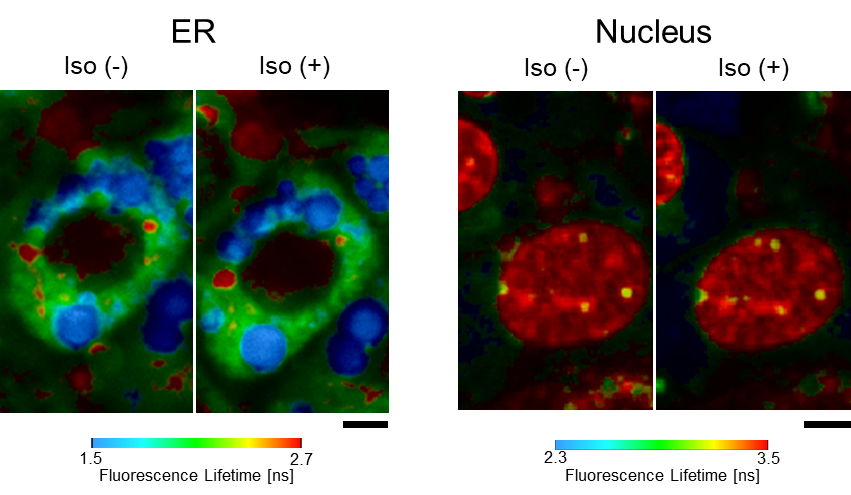


Figure S9. Thermal imaging of heat production in brown adipocytes using FLIM. FLIM analysis of heat production at ER and Nucleus. These data were relevant to Figure 3 in the main manuscript. Scale bar is 5 μm.

Experimental Procedures

General information: All the chemicals and solvents were purchased from Sigma Aldrich, Alfa Aesar, MERCK, Acros, TCI, Combi-blocks or Samchun and used without further purification. Normal phase purifications were carried out using Merck Silica Gel 60 (particle size: 0.040-0.063 mm, 230-400 mesh). HPLC purification was performed on Prep. HPLC (Shimazu) with a PDA detector. Purification method, unless indicated, gradient solvent system was water: acetonitrile (ACN) (90:10 to 0:100) with 0.1% formic acid in run time of 60 min; C18(2) Luna column (5 μm 100A, 250 × 21.2 mm) were used for purification. 1H-NMR and 13C-NMR spectra were recorded on a Bruker Avance 500 MHz and 850 MHz NMR spectrometer. Chemical shifts were expressed in parts per million (ppm). All photo-physical studies were performed in SpectraMax®M2e spectrophotometer (Molecular Devices) instrument and the obtained data were analyzed using Microsoft Office Excel and Origin 8.5.

**Synthesis**

BDN and ETG were synthesized as previously reported.[1,2]

**Scheme S1.** Synthetic scheme of DTG.

BDN (15 mg, 48.2 μmol) was dissolved in 5 mL dichloromethane and cooled to 0 °C, followed by addition of undecanoyl chloride (31.9 μL, 144.6 μmol) and triethylamine (40.3 μL, 289.2 μmol). The reaction was performed at 0 °C for 10 min, then moved to room temperature for 1 hour. The reaction mixture was concentrated under vacuum and purified by Prep. HPLC to get DTG (21.1 mg, 91.3 %). ^1^H NMR (850 MHz, Chloroform-*d*) δ 7.68 (d, J = 7.7 Hz, 3H), 7.48 (s, 1H), 7.31 (s, 2H), 6.44 (d, J = 2.9 Hz, 1H), 6.38 (s, 1H), 6.15 (s, 1H), 2.64 (s, 3H), 2.41 (t, J = 7.6 Hz, 2H), 1.77 (p, J = 7.5 Hz, 2H), 1.61 (s, 3H), 1.41 (p, J = 7.2 Hz, 2H), 1.38 – 1.34 (m, 2H), 1.31 (dd, J = 18.3, 11.6 Hz, 10H), 0.90 (t, J = 7.1 Hz, 3H). ^13^C NMR (214 MHz, Chloroform-*d*) δ 171.74, 161.94, 146.91, 143.01, 139.36, 138.56, 134.75, 133.60, 129.68, 129.31, 127.00, 123.26, 119.20, 116.08, 37.88, 31.89, 29.58, 29.50, 29.40, 29.31, 25.56, 22.68, 15.34, 15.16, 14.12. LC-MS m/z, calculated for [M+H]^+^: 480.3. Found: 480.2.

**Scheme S2.** Synthetic scheme of MTG.

Compound A1 was synthesized as previously reported.[3] BDN (14.3 mg, 45.9 μmol) was dissolved in 5 mL DMF. Compound A1 (14.6 mg, 30.6 μmol), HATU (12.8 mg, 33.7 μmol) and diethylisopropylamine (11.72 μL, 67.3 μmol) were dissolved in 1 mL DMF and then added to the solution. The reaction was stirred at room temperature for 12 hours. The reaction mixture was concentrated under vacuum and purified by Prep. HPLC to get MTG (16.9 mg, 47.8 %). ^1^H NMR (850 MHz, Methanol-*d*_4_) δ 7.91 – 7.88 (m, 3H), 7.85 – 7.81 (m, 6H), 7.77 (td, J = 7.9, 3.5 Hz, 6H), 7.73 (d, J = 8.4 Hz, 2H), 7.63 (s, 1H), 7.36 (d, J = 8.5 Hz, 2H), 6.44 (d, J = 3.6 Hz, 1H), 6.42 (dd, J = 3.8, 2.0 Hz, 1H), 6.29 (s, 1H), 3.49 (td, J = 13.6, 8.3 Hz, 2H), 2.58 (s, 3H), 2.51 (t, J = 7.1 Hz, 2H), 1.98 (p, J = 7.2 Hz, 2H), 1.81 (h, J = 8.3 Hz, 2H), 1.64 (s, 3H). ^13^C NMR (214 MHz, Methanol-*d*_4_) δ 172.25, 162.15, 147.10, 143.28, 139.97, 138.01, 134.93, 134.92, 134.58, 133.43, 133.39, 130.18, 130.12, 129.37, 129.11, 126.36, 123.10, 119.31, 118.65, 118.25, 115.62, 35.28, 26.03, 25.94, 21.67, 21.65, 21.33, 21.08, 14.05, 13.67. LC-MS m/z, calculated for [M]^+^: 656.3. Found: 656.2.

**Scheme S3.** Synthetic scheme of BDN-COOH.

BDN (100 mg, 321.4 μmol) was dissolved in 50 mL ACN, followed by addition of succinic anhydride (35.4 mg, 353.5 μmol). The solution was refluxed for 12 hours. The reaction mixture was concentrated under vacuum and purified by Prep. HPLC to get BDN-COOH (126.1 mg, 92.0 %). ^1^H NMR (850 MHz, Acetonitrile-*d*_3_) δ 8.68 (s, 1H), 7.76 (d, J = 8.1 Hz, 2H), 7.65 (s, 1H), 7.37 (d, J = 8.1 Hz, 2H), 6.49 (s, 1H), 6.43 (s, 1H), 6.30 (s, 1H), 2.67 (d, J = 11.9 Hz, 4H), 2.58 (s, 3H), 1.63 (s, 3H). ^13^C NMR (214 MHz, Acetonitrile-*d*_3_) δ 173.62, 170.80, 162.50, 147.70, 143.60, 140.37, 137.83, 134.56, 133.62, 129.70, 128.44, 126.66, 123.56, 118.75, 116.00, 31.21, 28.43, 14.56, 14.33. LC-MS m/z, calculated for [M+H]^+^: 412.2. Found: 412.2.

**Scheme S4.** Synthetic scheme of LTG.

4-(2-Aminoethyl)morpholine (4.8 μL, 36.5 μmol) was dissolved in 5 mL DMF. BDN-COOH (10 mg, 24.3 μmol), HATU (9.2 mg, 24.3 μmol) and diethylisopropylamine (8.5 μL, 48.6 μmol) were dissolved in 1 mL DMF and then added to the solution. The reaction was stirred at room temperature for 12 hours. The reaction mixture was concentrated under vacuum and purified by Prep. HPLC to get LTG (7.4 mg, 58.0 %). ^1^H NMR (850 MHz, Chloroform-*d*) δ 8.63 (s, 1H), 8.16 (s, 1H), 7.69 (s, 1H), 7.66 (d, J = 7.9 Hz, 2H), 7.32 (d, J = 7.9 Hz, 2H), 6.42 (d, J = 2.8 Hz, 1H), 6.40 – 6.36 (m, 1H), 6.15 (s, 1H), 4.02 – 3.98 (m, 4H), 3.78 – 3.73 (m, 2H), 3.71 (d, J = 10.6 Hz, 2H), 3.34 – 3.28 (m, 2H), 2.94 (s, 2H), 2.84 – 2.78 (m, 2H), 2.72 – 2.67 (m, 2H), 2.64 (s, 3H), 1.60 (s, 3H). ^13^C NMR (214 MHz, Chloroform-*d*) δ 174.27, 171.09, 162.08, 161.57, 161.39, 146.87, 142.82, 139.08, 138.60, 134.70, 133.59, 129.72, 129.63, 126.85, 123.30, 119.68, 116.08, 63.59, 57.98, 53.03, 33.70, 32.62, 31.17, 15.25, 15.17. LC-MS m/z, calculated for [M+H]^+^: 524.3. Found: 524.2.

**Scheme S5.** Synthetic scheme of PTG.

N-Boc-ethylenediamine (100 μL, 632 μmol), 5-chloro-1-pentyne (133.9 μL, 1264 μmol), potassium carbonate (698.7 mg, 5056 μmol), and potassium iodide (419.6 mg, 2528 μmol) were dissolved in 12 mL DMF. The reaction was refluxed for 48 hours. The solution was filtered and concentrated under vacuum to obtain A3. ^1^H NMR (500 MHz, Chloroform-*d*) δ 4.97 (s, 1H), 3.12 (d, J = 5.5 Hz, 2H), 2.46 (q, J = 6.6 Hz, 6H), 2.19 (td, J = 7.0, 2.6 Hz, 4H), 1.92 (t, J = 2.6 Hz, 2H), 1.60 (p, J = 6.9 Hz, 4H), 1.40 (s, 9H). ^13^C NMR (126 MHz, Chloroform-*d*) δ 156.00, 84.07, 78.88, 68.61, 53.46, 52.38, 38.34, 28.39, 25.88, 16.07. LC-MS m/z, calculated for [M+H]^+^: 293.2. Found: 293.2.

A3 (30.6 mg, 104.6 μmol) was dissolved in 20% TFA/DCM and stirred at room temperature for 2 hours. The reaction mixture was then evaporated and re-dissolved in 5 mL DMF. BDN-COOH (21.5 mg, 52.3 μmol), HATU (19.9 mg, 52.3 μmol) and diethylisopropylamine (91.1 μL, 523 μmol) were dissolved in 1 mL DMF and then added to the solution. The reaction was stirred at room temperature for 12 hours. The reaction mixture was concentrated under vacuum and purified by Prep. HPLC to get BDN-Alkyne (15.7 mg, 51.2 %). ^1^H NMR (850 MHz, Chloroform-*d*) δ 8.58 (s, 1H), 8.03 (s, 1H), 7.69 (d, J = 8.8 Hz, 3H), 7.31 (d, J = 8.3 Hz, 2H), 6.43 (d, J = 3.6 Hz, 1H), 6.38 (dd, J = 3.8, 2.0 Hz, 1H), 6.15 (s, 1H), 3.70 (q, J = 5.4 Hz, 2H), 3.39 – 3.32 (m, 4H), 3.30 (s, 2H), 2.83 – 2.79 (m, 2H), 2.70 – 2.66 (m, 2H), 2.63 (s, 3H), 2.36 (td, J = 6.5, 2.5 Hz, 4H), 2.07 (t, J = 2.6 Hz, 2H), 1.98 (s, 4H), 1.60 (s, 3H). ^13^C NMR (214 MHz, Chloroform-*d*) δ 174.98, 170.96, 162.09, 146.92, 142.90, 139.22, 138.55, 134.69, 133.60, 129.63, 129.56, 126.89, 123.32, 119.55, 116.06, 81.04, 71.00, 55.34, 52.98, 35.30, 32.59, 31.21, 22.11, 15.77, 15.32, 15.17.

Compound A4 was synthesized as previously reported.[4] CuSO_4_·5H_2_O (5 mg) and sodium ascorbate (5 mg) were dissolved in 300 μL H_2_O and wait the color to be yellow. To a 5 mL DMF solution of BDN-Alkyne (15.7 mg, 26.8 μmol) and A4 (46.2 mg, 64.3 μmol), the aqueous mixture was added. The reaction was stirred at 60℃ for 12 hours. The reaction mixture was concentrated under vacuum and purified by Prep. HPLC to get PTG (24.6 mg, 65.7 %). ^1^H NMR (850 MHz, Chloroform-*d*) δ 10.20 (s, 1H), 8.12 (s, 1H), 7.94 (s, 2H), 7.85 (s, 2H), 7.63 (s, 1H), 7.22 (s, 2H), 6.41 – 6.29 (m, 2H), 6.13 (s, 1H), 4.49 (s, 4H), 3.65 (s, 4H), 3.46 (d, J = 54.2 Hz, 6H), 3.26 (s, 4H), 3.11 (s, 8H), 2.89 (d, J = 64.9 Hz, 8H), 2.73 (d, J = 50.5 Hz, 6H), 2.59 (s, 5H), 2.48 (s, 4H), 2.13 (d, J = 20.4 Hz, 4H), 1.96 (s, 4H), 1.67 (s, 4H), 1.56 (s, 3H), 1.31 – 1.26 (m, 12H), 1.23 (d, J = 12.3 Hz, 24H), 0.87 (t, J = 7.2 Hz, 6H). LC-MS m/z, calculated for [(M+2H)/2]^+^: 698.0. Found: 698.0.

**Scheme S6.** Synthetic scheme of GTG.

D-sphingosine (7.3 mg, 24.3 μmol) was dissolved in 5 mL DMF. BDN-COOH (12 mg, 29.2 μmol), HATU (11.1 mg, 29.2 μmol) and diethylisopropylamine (10.2 μL, 58.4 μmol) were dissolved in 1 mL DMF and then added to the solution. The reaction was stirred at room temperature for 12 hours. The reaction mixture was concentrated under vacuum and purified by Prep. HPLC to get GTG (10.6 mg, 63.0 %). ^1^H NMR (850 MHz, Chloroform-*d*) δ 7.74 – 7.67 (m, 3H), 7.31 (d, J = 7.9 Hz, 2H), 6.42 (s, 1H), 6.38 (s, 1H), 6.14 (s, 1H), 5.87 – 5.79 (m, 1H), 5.55 (dd, J = 15.3, 5.7 Hz, 1H), 4.40 (s, 1H), 4.03 – 3.98 (m, 1H), 3.98 – 3.93 (m, 1H), 3.77 (d, J = 10.0 Hz, 1H), 2.85 (s, 2H), 2.75 (s, 2H), 2.64 (s, 3H), 2.07 (q, J = 6.8 Hz, 2H), 1.60 (s, 3H), 1.41 – 1.36 (m, 2H), 1.33 – 1.25 (m, 22H), 0.90 (t, J = 7.1 Hz, 3H). ^13^C NMR (214 MHz, Chloroform-*d*) δ 173.18, 171.23, 162.02, 146.81, 142.84, 139.13, 138.63, 134.70, 134.39, 133.57, 129.69, 128.30, 126.93, 123.26, 119.41, 116.07, 74.17, 61.81, 54.89, 32.84, 32.32, 31.93, 31.44, 29.70, 29.69, 29.66, 29.63, 29.50, 29.36, 29.25, 29.13, 22.69, 15.32, 15.16, 14.12. LC-MS m/z, calculated for [M-F]^+^: 693.4. Found: 673.4.

**Scheme S7.** Synthetic scheme of NTG.

BDN-COOH (11.3 mg, 27.5 μmol), N-hydroxysuccinimide (4.7 mg, 41.2 μmol) and N-(3-Dimethylaminopropyl)-N’-ethylcarbodiimide hydrochloride (7.9 mg, 41.2 μmol) were dissolved in 3 mL DMF. The reaction was stirred at room temperature for 12 hours. The resulted crude BDN-COOH-NHS was then used directly for next step.

Compound A2 was synthesized as previously reported.[5] A2 (18.3 mg, 30.7 μmol) was dissolved in 20% TFA/DCM and stirred at room temperature for 2 hours. The reaction mixture was then evaporated and re-dissolved in 1 mL DMF, followed by addition of BDN-COOH-NHS and diethylisopropylamine (47.9 μL, 275 μmol). The reaction was stirred at room temperature for 12 hours. The reaction mixture was concentrated under vacuum and purified by Prep. HPLC to get NTG (10.6 mg, 43.3 %). ^1^H NMR (850 MHz, Methanol-*d*_4_) δ 8.33 (s, 1H), 8.26 (s, 1H), 8.07 (t, J = 5.5 Hz, 1H), 8.03 (d, J = 8.6 Hz, 2H), 7.98 – 7.94 (m, 1H), 7.73 (d, J = 8.3 Hz, 2H), 7.70 (d, J = 8.3 Hz, 1H), 7.58 (s, 1H), 7.55 (d, J = 8.6 Hz, 1H), 7.26 (d, J = 8.4 Hz, 2H), 7.24 – 7.19 (m, 1H), 7.10 (dd, J = 8.7, 2.0 Hz, 1H), 7.07 (d, J = 8.7 Hz, 2H), 6.40 (d, J = 3.4 Hz, 1H), 6.36 (dd, J = 3.5, 1.9 Hz, 1H), 6.13 (s, 1H), 4.06 (t, J = 6.4 Hz, 2H), 3.44 (s, 4H), 3.37 (d, J = 4.7 Hz, 4H), 3.30 (q, J = 6.6 Hz, 2H), 2.91 (s, 3H), 2.74 (t, J = 6.9 Hz, 2H), 2.60 (t, J = 6.9 Hz, 2H), 2.47 (s, 3H), 1.89 – 1.83 (m, 2H), 1.76 – 1.70 (m, 2H), 1.54 (s, 3H). ^13^C NMR (214 MHz, Methanol-*d*_4_) δ 174.64, 173.12, 167.22, 163.35, 162.62, 155.43, 154.09, 148.41, 148.40, 139.23, 135.93, 134.73, 130.69, 130.20, 129.61, 127.76, 125.16, 124.38, 122.77, 120.60, 116.93, 116.73, 116.13, 103.40, 68.88, 55.23, 50.13, 44.01, 40.05, 33.10, 31.88, 27.57, 27.03, 15.42, 14.99. LC-MS m/z, calculated for [M+H]^+^: 889.4. Found: 889.4.

**References**

[1] N. Y. Kang, S. C. Lee, S. J. Park, H. H. Ha, S. W. Yun, E. Kostromina, N. Gustavsson, Y. Ali, Y. Chandran, H. S. Chun, M. A. Bae, J. H. Ahn, W. Han, G. K. Radda, Y. T. Chang, Visualization and Isolation of Langerhans Islets by a Fluorescent Probe PiY. Angew. Chem. Int. Ed. 52 (2013) 8557−8560.

[2] D. Su, J. Oh, J. C. Lee, J. C. Lim, S. Sahu, X. Yu, D. Kim, Y. T. Chang, Dark to light! A new strategy for large Stokes shift dyes: coupling of a dark donor with tunable high quantum yield acceptors. Chem. Sci. 5 (2014) 4812−4818.

[3] D. J. Weix, S. D. Dreher, T. J. Katz, [5]HELOL Phosphite:  A Helically Grooved Sensor of Remote Chirality. J. Am. Chem. Soc. 122 (2000) 10027−10032.

[4] M. Collot, R. Kreder, A. L. Tatarets, L. D. Patsenker, Y. Melya, A. S. Klymchenko, Bright fluorogenic squaraines with tuned cell entry for selective imaging of plasma membrane vs. endoplasmic reticulum. Chem. Commun. 51 (2015) 17136−17139.

[5] G. Lukinavičius, C. Blaukopf, E. Pershagen, E. Schena, L. Reymond, E. Derivery, E. Gonzalez-Gaitan, E. D’Este, S. W. Hell, D. W. Gerlich, K. Johnsson, SiR-Hoechst is a far-red DNA stain for live-cell nanoscopy. Nat. Commun. 6 (2015) 8497.

[6] S. Arai, S. C. Lee, D. Zhai, M. Suzuki, Y. T. Chang, A Molecular Fluorescent Probe for Targeted Visualization of Temperature at the Endoplasmic Reticulum. Sci. Rep. 4 (2014) 6701.
